# Orofacial Clefts: Genetics of Cleft Lip and Palate

**DOI:** 10.3390/genes14081603

**Published:** 2023-08-09

**Authors:** Arwa Babai, Melita Irving

**Affiliations:** Department of Clinical Genetics, Guy’s Hospital, Guy’s and St Thomas’ NHS Foundation Trust, London SE1 9RT, UK; melita.irving@gstt.nhs.uk

**Keywords:** cleft lip, cleft palate, syndromic orofacial clefts

## Abstract

Orofacial clefting is considered one of the commonest birth defects worldwide. It presents as cleft lip only, isolated cleft palate or cleft lip and palate. The condition has a diverse genetic background influenced by gene–gene and gene–environment interaction, resulting in two main types, syndromic and nonsyndromic orofacial clefts. Orofacial clefts lead to significant physiological difficulties that affect feeding, speech and language development and other developmental aspects, which results in an increased social and financial burden on the affected individuals and their families. The management of cleft lip and palate is solely based on following a multidisciplinary team approach. In this narrative review article, we briefly summarize the different genetic causes of orofacial clefts and discuss some of the common syndromes and the approach to the management of orofacial clefts.

## 1. Introduction

One of the most complicated processes in the human body is craniofacial development. It involves a combination of complex interactions between molecular signals and transcription factors that patterns the morphogenesis of the craniofacial organs, with the cranial neural crest cells (CNCs) playing an important key role [1]. Disruption early on during embryogenesis, between 4 to 12 weeks, could result in the failure of the proper fusion of the facial prominences, resulting in congenital anomalies such as oral clefts. Orofacial clefts (OFCs), secondary to disruption in this signaling network, are among the most common congenital anomalies, affecting around 1 in 700 live births worldwide, and this prevalence varies significantly between populations [2,3].

## 2. Methods

For this article, we carried out a narrative review focusing on the genetics of cleft lip and palate for both syndromic and nonsyndromic types, and briefly highlight the embryological mechanisms behind each type. We also discuss a few examples of the most common syndromes and the multidisciplinary management approach for orofacial clefts. Relevant literature published up to 2023 was reviewed using mainly the PubMed, Science Direct, OMIM and Google Scholar electronic databases. The search process involved using the following keywords: “Embryology”, “Cleft + Lip”, “Cleft + Palate”, “Cleft + lip + and + palate” and “Non-syndromic + orofacial + clefts”, all combined into different combinations using Boolean operators (AND, OR).

## 3. The Embryonic Development of the Face and Orofacial Clefts

Early on, during the third week of embryonic life, the embryo resembles a flat disc plate made of three layers of pluripotent germ cells: the ectoderm, mesoderm and endoderm. The development of the human face takes place between the fourth and the twelfth week of embryonic development, originating from the 1st and 2nd pharyngeal arches [4]. The complex orofacial morphogenesis process was described in detail by Y. Ji et al. [5] Neural crest cells (NCCs) originating from the ectodermal layer proliferate and form the neural tube. The rapid growth of the neural cells results in the expansion of the cranial region and elongation and folding of the neural tube in week 4 of embryonic development, and the development of the face from five primordia or facial prominences [6]. These comprise the following: the frontonasal prominence, which forms the forehead, the nose and the top of the primitive mouth; the two maxillary prominences that will form the lateral part of the upper lip; and the two mandibular prominences that form the lower lip and the lower jaw. This process is followed by the development of the medial nasal process (MNP) and lateral nasal process (LNP) from the nasal placode.

Facial development is a complex process that includes the development of the mouth, lips, palate and nose, and it takes place between the fourth and the twelfth week of embryonic development. A series of cellular growth and differentiation, migration and apoptosis takes place in a highly coordinated manner [7], and defects in this sequence of events could result in the development of cleft lip, cleft palate or both, and, although cleft lip and palate occur together, their embryonic origin is different.

### 3.1. Embryology of Cleft Lip

Development of the lip takes place between the 4th and the 8th week of gestation. During this period, several events take place to contribute to the development of the lip. At the beginning, the maxillary prominences fuse with the lateral nasal prominence, forming the lateral parts of the upper lip, and the lateral nasal prominence forms the alae of the nose [8]. The continuous growth of the maxillary prominence medially and its eventual fusion with the medial nasal prominence form the nostrils. The fusion of the medial nasal prominences forms the structures of the nose and the philtrum. The fusion between the maxillary prominence and the medial nasal prominence also forms a mass of mesenchymal tissue, which, as it continues to grow, separates the upper lip from the nostrils, forming the primary palate during the 7th week [9]. Any delay or alteration in this process caused by the failure of the fusion between the maxillary prominence and the nasal prominence on one side will result in a unilateral cleft lip. Bilateral failure of the fusion between these two prominences leads to a bilateral cleft lip. The exact molecular pathway that results in cleft lip and palate remains a target for multiple molecular studies [10,11].

### 3.2. Embryology of Cleft Palate

The development of the palate begins during the fifth week of gestation, and continues until the twelfth gestational week. The most critical time during palate development is between the sixth and the nineth gestational week. During the sixth week, two lateral palatine processes or the palatal shelves grow from the medial side of the maxillary prominence and lie vertically under the tongue [4,12]. When the tongue starts to flatten and move inferiorly as a result of the development of the jaw, the two palatal shelves start to elevate to a horizontal position and grow approaching each other [9]. The palatal shelves then fuse with each other and with the nasal septum and the hard palate. The fusion is completed by the twelfth week to form the hard palate from the fusion of the bones extending from the maxilla and the palatine bone to the palatal shelves, while the posterior unossified part forms the soft palate and the uvula [12,13]. Failure of the elevation, contact or fusion of the palatal shelves results in clefts.

## 4. Signaling Pathways in Orofacial Clefts

Signaling molecules involving multiple signaling pathways such as WNT, TGF/BMP and FGF and morphogens have been studied for their involvement in the pathogenesis of cleft lip, and cleft lip and palate.

### 4.1. WNT Pathway

Studies have suggested that the WNT signaling pathway includes canonical and noncanonical modes of signaling and plays a key role in facial morphogenesis by regulating crucial and critical processes for lip and palate development, such as cell proliferation, migration and differentiation, and also cross-interact with other pathways [10]. Disruption in this pathway dysregulates the normal developmental pathways leading to cleft lip and/or cleft palate development of both the syndromic and nonsyndromic type [11]. Pathogenic variants in the genes involved in the WNT pathway such as the *Wnt3A* gene were found to be associated with nonsyndromic cleft lip/or palate [14] and could also affect the development and morphogenesis of the neural crest, leading to the development of orofacial clefts. The WNT pathway is also implicated in the development of other diseases such as cancer [15] and skeletal disorders [16].

### 4.2. TGF-β Signaling Pathway

The TGF-β (Transforming Growth Factor-β) signaling pathway plays a crucial role in various biological and cellular processes regulating cell growth, immune responses, embryonic development and other processes [17]. In facial morphogenesis, TGF-β signaling is essential for palatal fusion through its interaction with other signaling pathways such as WNT, FGF and BMP. TGF-β is involved in epithelia mesenchymal transition, a vital step for successful palatal shelves migration and fusion [18]. The TGF-β signaling pathway involves several genes, and pathogenic variants in some of these genes have been shown to be associated with the development of orofacial clefts, such as the variants in the Interferon Regulatory Factor 6 (*IRF6*) gene associated with Van der Woude syndrome (VWS) [3]; the SMAD gene family, which also cross-interacts with the BMP signaling pathway; and variants in these genes are associated with an increased risk of cleft lip development [18].

### 4.3. BMP Signaling Pathway

The BMP (Bone Morphogenetic Protein) signaling pathway regulates cell proliferation, cell differentiation and apoptosis, which are critical steps for facial morphogenesis [19,20]. The BMP pathway influences the regulation of certain genes involved in palatal fusion and interacts with other cellular pathways such as the SHH signaling pathway [21], which is also important for craniofacial development. BMP2 and BMP4 are members of the BMP protein family. BMP2 is crucial for the development of the facial processes during craniofacial morphogenesis, while BMP4 is important for tissue differentiation and the formation of the facial prominences [22]. Dysregulation in these processes results in cleft lip or cleft palate [23].

## 5. Epidemiology of Cleft Lip and Palate

**Figure 1:**
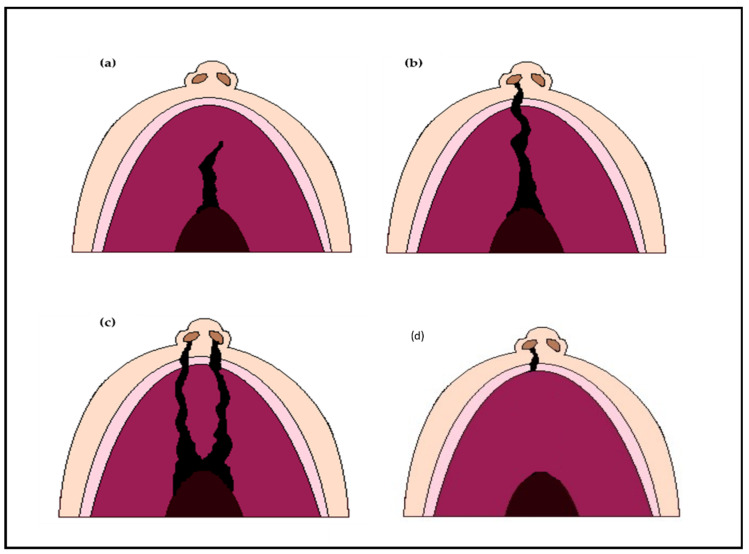
Types of orofacial clefts; (**a**) Incomplete cleft palate only; (**b**) Unilateral complete cleft lip and palate; (**c**) Bilateral complete cleft lip and palate; (**d**) Cleft lip only.

Oral clefts are divided into different types, including cleft lip (CL), complete or incomplete cleft palate (CP) and unilateral or bilateral cleft lip and palate (CLP) [Figure 1]. The global prevalence of oral clefts has been estimated to be around 0.45 in 1000 live births, though it is noted to be variable among different populations, possibly owing to variable environmental and socio-economic factors [24]. The prevalence is as high as 1 in 500 live births in the Asian population and drops to 1 in 2500 in the African population [2].

In developed countries, orofacial malformations significantly impact the quality of life and place an enormous psychosocial burden on affected individuals prior to treatment or among affected adults who are dissatisfied with their facial appearance. The management of orofacial clefts also places a significant economic burden on the healthcare system and the whole society [25].

Individuals with CLP experience other consequences besides facial deformity. Feeding difficulties in infants could lead to failure to thrive. Left untreated, CLP could also affect speech development and in some cases it could be associated with hearing loss and dental malocclusion. Therefore, best practice involves evaluation by a multidisciplinary team including a pediatrician, clinical geneticist, speech and language therapist, feeding and nutrition specialist, social worker and psychologist in order to cover the different aspects associated with CLP and provide optimum management [26].

In developed countries, CLP is not considered as a major cause of increased mortality; however, recent studies have suggested that the lifespan of individuals affected with orofacial clefts is shorter, and the overall risks for most of the major causes of death is higher in comparison to the general population [27]. Studies have observed a significantly increased risk of suicide and a marginally increased risk of cancer-related mortality in patients with orofacial clefts [27,28]. Another study noticed a higher occurrence of primary brain cancer and breast cancer in females suffering from orofacial clefts, while male individuals with cleft lip/or palate showed an increased occurrence of primary lung cancer [29].

## 6. Factors Leading to Orofacial Clefting

Multiple factors have been found to contribute to the development of orofacial clefts. Clefts could be of the syndromic type and develop as part of Mendelian syndromes, or they could develop as part of the clinical phenotype associated with a chromosomal anomaly. Also, prenatal exposure to certain teratogens and environmental factors has been proved to be associated with the development of orofacial clefts.

### 6.1. Genetics Evidence

The complex process of craniofacial development and the different strategies used to understand its mechanism has been demonstrated in different studies [9,30]. The evidence of underlying genetic factors influencing the risk of developing oral cleft was supported by twin studies [31,32]. An orofacial cleft population-based cohort study has shown that the relative recurrence risk for CL was 32, and CP was found to be 65 times higher among first-degree relatives [33].

With the recent advances in genomic technology, many studies have revealed several different molecular mechanisms implicated in the cause of syndromic and nonsyndromic orofacial clefts [34,35], improving understanding of how the molecular networks interact with other genetic pathways.

### 6.2. Environmental Factors

Environmental factors are also known to play a crucial role in nonsyndromic cleft lip and palate pathogenesis through interactions with different genes in susceptible individuals [36]. For example, several studies have shown that the association is significant between maternal smoking and nonsyndromic orofacial clefts [37,38,39], which is also applicable to passive smoking [40]. However, this association is not strong, as studies have shown that the risk for orofacial clefts secondary to smoking during pregnancy is minor, with an odds ratio of around 1.3 for CLP [41]. However, this effect is amplified in the presence of increased genetic susceptibility [42,43]. This phenomenon was evident in smoking mothers with fetuses lacking active forms of glutathione s-transferase GSTT1 and GSTM1, creating a seven-fold increased risk of orofacial clefts [44].

Alcohol has a well-recognized teratogenic effect during pregnancy and it is associated with causing fetal alcohol syndrome. Alcohol exposure increases the risk of orofacial clefts, as shown in previous studies (CL with or without CP odds ratio = 2.2, CP odds ratio = 2.6), and this risk was found to be dose-dependent [45]. It is not known how this association is influenced by genetic variations in alcohol metabolism. One study proposed that variants in alcohol dehydrogenase 1C (*ADH1C*) involved in alcohol metabolism might be associated with and have an effect on the risk of clefts in fetuses through its role in alcohol metabolism [46]. Yet, the exact mechanism remains to be demonstrated.

Studies have well described the preventive role of folic acid in the prevention of birth defects like neural tube defects [47]. Multiple studies have also evaluated the association between the development of orofacial clefts and folic acid supplements [48,49,50] and supported the protective effect that a high dose of folic acid supplement has in significantly reducing the risk of CLP [50,51]. Studies have also looked for possible gene–environment interaction effects between folic acid and the transforming growth factor α gene (*TGFA*) to further understand the association with orofacial cleft risk. TGFa is a secretory protein encoded by *TGFA*, one of the well-studied candidate genes for OFC that is highly expressed in the palatal shelves epithelia and plays a key role in palatal fusion [52]. The effect of genetic polymorphisms in *TGFA* in combination with folic acid deficiency within the first trimester has been investigated, and babies homozygous to *TGFA* TaqI A2 allele genotype were found to have 3 to 8 times increased risk of orofacial clefts [53,54]. *TGFA* variants were also found to lead to a ten-fold rise in the risk of orofacial clefts when joined with pathogenic variants in the *MSX1* gene, giving an example of gene–gene interaction [55]. Since ethnicity is associated with variations in genotype, this could suggest a variable effect of gene–environment interaction between different ethnic groups and could explain the some of the geographic and ethnic variation on orofacial clefts prevalence and epidemiology [56].

## 7. Nonsyndromic Orofacial Clefts

Nonsyndromic cleft lip or cleft palate is an isolated condition with complex genetically heterogenous backgrounds, and is not associated with any other obvious anomalies. Around 70% of the cases of cleft lip and/or cleft palate are of the nonsyndromic type. It is also estimated that around half of the cases of cleft palate only (CPO)are nonsyndromic [35]. Despite the growth in our understanding of the genetic etiology of syndromic orofacial clefts by the identification of many monogenic syndromes, which has been assisted by the rapid advance in genomics and genetics technology, the progress in our understanding of the genetic etiology of nonsyndromic orofacial clefting remains relatively slow in comparison to the syndromic type [57]. This could be explained by the genetic heterogenicity of nonsyndromic orofacial clefts, the contribution of environmental factors and gene–environment interaction, and its deviation from the Mendelian inheritance, as most of the cases seems to be sporadic, despite the compelling evidence of a genetic component collected from twin studies. However, this is also improving now with the current rapid development of genomic tools and genome sequencing technologies.

### 7.1. Genetic Studies of Nonsyndromic Cleft Lip with/without Cleft Palate

Many studies have looked into the identification of genetic loci and candidate genes associated with orofacial clefts adopting different approaches.

### 7.2. Linkage Studies

Linkage studies are among the powerful gene-hunting tools used to study the genetic etiology of nonsyndromic orofacial clefts. Multiplex families or multiple affected relatives are required to conduct these studies, as the principle of linkage studies is based on identifying regions in the genome that possess a disease-causing gene or locus by assessing the segregation of DNA markers in related individuals to see whether or not these markers co-segregate with the disease phenotype. The genetic complexity of nonsyndromic orofacial clefts limited the success of linkage studies, and these were extended to cover the whole genome, becoming known as genome-wide linkage studies [58]. Genome-wide linkage studies were able to identify novel and known candidate genes for nonsyndromic cleft lip and/or palate, such as *LPHN2* at 1p31, *PVRL3* at 3q13.3 and several other loci in a study by Mohamad Shah et al. [59]. Candidate regions have been reported on chromosome 2p24-p25, 1q32-q42 and 2p24-p25 [60] 18q21.1 [61] and chromosome 1q32.2-41 [60]. Despite the contribution of linkage studies to identifying candidate chromosomal regions, those regions are wide, which makes it difficult to identify the specific locus or disease-causing gene; hence the need to adopt other genomic approaches.

### 7.3. Candidate Gene Approach

Unlike linkage studies, candidate gene studies are targeted genetic analysis studies that depend on isolated cases and not multiple affected individuals in a family. This approach looks for a statistically significant association between an allele and the specific phenotype under interest and the allele frequency variation between affected individuals with this phenotype and unaffected individuals. The candidate gene approach helps to identify the causative variant, and the selection of the candidate genes to study depends on having previous knowledge about the molecular pathways and interaction networks involved in the disease mechanism [62] by selecting the genes known to be involved in facial development and other relevant pathways.

The Interferon Regulatory Factor 6 (*IRF6*) gene is one of the earliest sustainability genes for nonsyndromic cleft lip identified [63] and proved to be the causative gene for Van der Woude syndrome. Genes such as *RYK*, *TBX22*, *FGFR1*, *NAT2*, *GSTT1* and others have also been found to be associated with NSCLP [64,65,66,67,68,69].

### 7.4. Genome-Wide Association Studies

Genome-wide association studies represent an important advance in genomic studies and high-throughput genomic tools are useful in detecting the possible genetic loci and genes contributing to nonsyndromic cleft lip/palate, taking into account the complexity of the genetic nature of nonsyndromic orofacial clefts and the contribution of other factors such as gene–environment and gene–gene interaction and testing the whole genome in search for associations.

The first nonsyndromic orofacial clefts GWAS was published in 2009 [70] showing a susceptibility locus in chromosome 8q24 associated with an increased risk of NSCL/P, which was replicated in other studies [71], while some studies highlighted a significant amount of evidence that ethnicity plays a role in determining susceptibility loci [72,73]. To date, studies have reported the association between more than 43 genes and loci with nonsyndromic orofacial clefts such as risk loci at chromosomal regions 1q, 9q, 16p12.1, 17q22 and others [74,75,76,77,78] and variants in susceptibility genes involved in craniofacial development such as *IRF6* gene [78], *MAFB* [79], *MAX1* [80] and *TGFA* [81].

## 8. Syndromic Orofacial Clefts

Orofacial clefts are divided into syndromic and nonsyndromic. Nonsyndromic orofacial clefts represent the majority of cases and are further divided into familial and sporadic types. Syndromic orofacial clefts are rare. With the recent advances in genetic testing capability, around 500 syndromes associated with cleft lip or palate have now been identified [82]. According to the underlying genetic cause, they are generally subdivided into Mendelian monogenic syndromes caused by single gene disorders, chromosomal syndromes caused by chromosomal abnormalities, teratogens and a group of unknown genetic syndromes.

## 9. Monogenic Syndromes

This group of syndromes is caused by pathogenic variants in single genes. Cohen published a review in 1978 in which he clinically described 154 of these syndromes [83]. However, with the development in molecular technology, extensive research has followed to identify the underlying genes and variants associated with these syndromes, and further syndromes have now been delineated.

### Common Monogenic Syndromes

Van der Woude syndrome (VWS; OMIM #119300) is the most common syndrome associated with orofacial clefts, accounting for 2% of all CLP cases [84]. Anne Van der Woude described the syndrome for the first time in 1954 [85]. VWS is a rare autosomal dominant syndrome of high penetrance and variable expression, associated with CL with or without CP or isolated CP and by lower lip pits or fistulae [86]. Two genes have so far been linked to VWS. In total, 70% of VWS cases are found to be due loss-of-function variants in Interferon Regulatory Factor 6 (*IRF6*) [87]. *IRF6* pathogenic variants were also detected in nonsyndromic cleft lip and palate [88] and popliteal pterygium syndrome (PPS; OMIM # 119500) [87,89]. *IRF6* produces a transcription factor protein that plays a vital role in craniofacial development during embryogenesis [90].

Dominant pathogenic variants in Grainyhead-like transcription factor 3 (*GRHL3*) were reported in 5% of the individuals with VWS and negative for pathogenic variants in *IRF6* [91]. A recent study has identified a new rare pathogenic variant in *NME1* and *NME2* genes and their NME1/NME1 protein complex in individuals with VWS and negative for pathogenic variants in both *IRF6* and *GRHL3*, suggesting that disruption in the IRF6-NME complex during lip morphogenesis leads to CLP, recommending further studies to evaluate *NME1* as a possible third cause of VWS [92].

Another example of a common monogenic syndrome is Stickler syndrome, a collagen-associated connective tissue disorder with autosomal dominant transmission, which has been clinically and genetically described in the literature [93]. It is a multisystemic disease predominantly associated with ocular features such as cataract, myopia of high grade and retinal detachment, auditory symptoms of hearing loss, orofacial clefts as cleft palate (could be part of Robin sequence) and articular manifestations such as joint hypermobility and epiphyseal dysplasia [94]. Stickler syndrome has a variable intrafamilial expression that could be explained by locus heterogeneity [95]. It is subclassified into type 1 (OMIM 108300) and type 2 (OMIM 184840). The systemic features are similar for both subgroups. Stickler syndrome results from mutations in one of six genes encoding the cartilage-expressed collagens II, IX and XI (*COL2A1*, *COL3A1*, *COL11A2*, *COL9A1*, *COL9A2*, *COL9A3*) [96].

Recent developments in next-generation sequencing technology have also helped in identifying the causative genes for other rare syndromes with orofacial clefts such as Miller syndrome [97], Kabuki syndrome [98] and others.

## 10. Chromosomal Syndromes

Velocardiofacial (Shprintzen) syndrome (OMIM 192430) is an autosomal dominant condition first detected by Shprintzen et al. in 1978 in twelve patients [99]. VCFS is characterized by cleft palate or submucosal CP, cardiac anomalies, dysmorphic facies, velopharyngeal insufficiency and learning and developmental disabilities [100,101]. VCFS is caused by a recurrent microdeletion on the long arm of chromosome 22 (del22q11.2), the same chromosome that was initially linked to DiGeorge syndrome (DGS; OMIM 188400), which is also due to deletions at 22q11.2, hence the clinical picture overlap [101], and is detected by either chromosomal microarray testing or fluorescence in situ hybridization (FISH).

Emanuel syndrome (OMIM 609029) is a chromosomal disorder characterized by failure to thrive, developmental delay, craniofacial anomalies as CP, preauricular pits, microcephaly, renal anomalies, congenital heart defects, male genital abnormalities, micrognathia and learning disability [102,103]. It is a rare disorder with unknown prevalence, caused by the presence of supernumerary derivative chromosome with chromosomal material from both chromosome 11 and 22 from an unaffected balanced translocation carrier parent [104].

Other chromosomal disorders underlying different syndromes characterized by orofacial clefts include trisomy 13, trisomy 18 and Wolf–Hirschhorn syndrome caused by a deletion involving chromosome 4p16.3 [105].

## 11. Genetic Counselling

Early evaluation with genetic testing and management is recommended for individuals and families affected with OFC to help with managing the emotional and psychological stress associated with this condition. Counselling is a very important part of the patient and family support.

Besides offering emotional support that helps in building a positive self-image and maintaining self-esteem, genetic counselling also involves discussing treatment options. Primary and secondary surgical correction and repair are the mainstay of OFC management. There is a wide variability in surgical treatment protocols, techniques and timing for cleft lip and palate repair between developed countries, despite the presence of recommendations by the WHO for cleft care [106], and only few randomized controlled trails have addressed cleft treatment [107].

Cleft care is based on a multidisciplinary team management approach including a pediatrician, psychologist, cleft surgeon, clinical geneticist, speech and language therapist, clinical nurse specialist, ear nose and throat surgeon, audiologist and orthodontist to help tackle the different complications associated with cleft lip and palate.

The prenatal diagnosis of CL and CP is now possible with the advance in antenatal imaging modalities and genetic testing, and prenatal diagnosis combined with counselling was found to play a crucial rule in preparing the family antenatally and improving the quality of care postnatally and the overall quality of life [108].

Recurrence risk varies between syndromic and nonsyndromic CL ± CP. Nonsyndromic CLP is a multifactorial trait caused by gene–environment interaction; hence the recurrence risk is influenced by different factors such as the clefting severity, the family history and number of affected individuals within the family, and the affected individual’s sex and whether or not this is the most affected sex [109]. For example, the recurrence risk for isolated bilateral CLP has been estimated at around 4.6% compared to 2.5% in individuals with unilateral cleft only [110].

## 12. Conclusions

The rapid developments in genetic testing techniques have facilitated a significant number of discoveries of candidate genes and described new syndromes, which have helped in increasing our understanding of the molecular mechanisms that underlie OFC development. Improvements in antenatal genetic diagnosis have allowed early intervention and management which have improved the affected individuals’ quality of life and overall quality of care. Further research is needed to look specifically at the role of the gene–environment interaction in causing CLP, in order to identify possible preventive measures.

## Data Availability

No new data were created or analyzed in this study. Data sharing is not applicable to this article.
